# SARS-CoV-2 exploits host DGAT and ADRP for efficient replication

**DOI:** 10.1038/s41421-021-00338-2

**Published:** 2021-10-26

**Authors:** Shuofeng Yuan, Bingpeng Yan, Jianli Cao, Zi-Wei Ye, Ronghui Liang, Kaiming Tang, Cuiting Luo, Jianpiao Cai, Hin Chu, Tom Wai-Hing Chung, Kelvin Kai-Wang To, Ivan Fan-Ngai Hung, Dong-Yan Jin, Jasper Fuk-Woo Chan, Kwok-Yung Yuen

**Affiliations:** 1 State Key Laboratory of Emerging Infectious Diseases, Carol Yu Centre for Infection, Department of Microbiology, Li Ka Shing Faculty of Medicine, The University of Hong Kong, Pokfulam, Hong Kong Special Administrative Region China; 2 Department of Clinical Microbiology and Infection Control, The University of Hong Kong-Shenzhen Hospital, Shenzhen, Guangdong Province China; 3 Department of Microbiology, Queen Mary Hospital, Pokfulam, Hong Kong Special Administrative Region China; 4 Department of Medicine, Li Ka Shing Faculty of Medicine, The University of Hong Kong, Pokfulam, Hong Kong Special Administrative Region China; 5 School of Biomedical Sciences, Li Ka Shing Faculty of Medicine, The University of Hong Kong, Pokfulam, Hong Kong Special Administrative Region China; 6 Academician workstation of Hainan Province of Hainan Medical University, and Hainan Medical University-The University of Hong Kong Joint Laboraotry of Tropical Infectious Diseasees, The University of Hong Kong, Pokfulam, Hong Kong Speical Administrative Region China

**Keywords:** Mechanisms of disease, Metabolomics

## Abstract

Coronavirus Disease 2019 (COVID-19) is predominantly a respiratory tract infection that significantly rewires the host metabolism. Here, we monitored a cohort of COVID-19 patients’ plasma lipidome over the disease course and identified triacylglycerol (TG) as the dominant lipid class present in severe acute respiratory syndrome coronavirus 2 (SARS-CoV-2)-induced metabolic dysregulation. In particular, we pinpointed the lipid droplet (LD)-formation enzyme diacylglycerol acyltransferase (DGAT) and the LD stabilizer adipocyte differentiation-related protein (ADRP) to be essential host factors for SARS-CoV-2 replication. Mechanistically, viral nucleo capsid protein drives *DGAT1*/*2* gene expression to facilitate LD formation and associates with ADRP on the LD surface to complete the viral replication cycle. *DGAT* gene depletion reduces SARS-CoV-2 protein synthesis without compromising viral genome replication/transcription. Importantly, a cheap and orally available DGAT inhibitor, xanthohumol, was found to suppress SARS-CoV-2 replication and the associated pulmonary inflammation in a hamster model. Our findings not only uncovered the mechanistic role of SARS-CoV-2 nucleocapsid protein to exploit LDs-oriented network for heightened metabolic demand, but also the potential to target the LDs-synthetase DGAT and LDs-stabilizer ADRP for COVID-19 treatment.

## Introduction

The collective scientific understanding of Coronavirus Disease 2019 (COVID-19) has evolved rapidly since its emergence, from the recognition of the causative virus, evaluation of therapeutics, to the development of multiple candidate vaccines within the span of a year^1–4^. However, there remain many significant unanswered questions, especially with regard to the underlying molecular mechanisms associated with the metabolic alterations of COVID-19^5^. It is still unclear what are the SARS-CoV-2 determinants reprogramming host metabolism and what are the essential host factors orchestrating the heightened metabolic demand during virus propagation.

To fulfill the requirements of rapid and massive clonal replication, viruses must co-opt distinct host programs to meet heightened metabolic demands. A key component in such reprogramming is the rapid upregulation of lipid biosynthesis, which builds up structural elements of double-membrane lipid vesicles for assembly of synthesized virus components including the lipid envelope. We previously documented the essentiality of lipogenic reshaping in Middle East respiratory syndrome coronavirus (MERS-CoV) replication^6^, which is another betacoronavirus that can cause life-threatening infections in humans. In COVID-19 patients, obesity increases the risk of severe disease^7^ and lipid droplets (LDs) fuel SARS-CoV-2 replication^8^. However, the precise mechanisms involved in these host–virus interactions remain largely mysterious.

In this study, we performed both plasma and cellular lipidomic profiling to compare SARS-CoV-2 and SARS-CoV infections. Our results identified triacylglycerol (TG) as the dominant lipid class of SARS-CoV-2-induced metabolic dysregulation. Further analysis along the TG synthetic pathways demonstrated that both diacylglycerol acyltransferase (DGAT) and the LD stabilizer adipocyte differentiation-related protein (ADRP) are potential antiviral targets for SARS-CoV-2 infection. Importantly, we showed that that Xanthohumol, a DGAT1/2 inhibitor with both antiviral and anti-inflammatory properties, may serve as an orally available treatment option for COVID-19.

## Results

### SARS-CoV-2 reprograms host TG metabolism upon infection

**Fig. 1:**
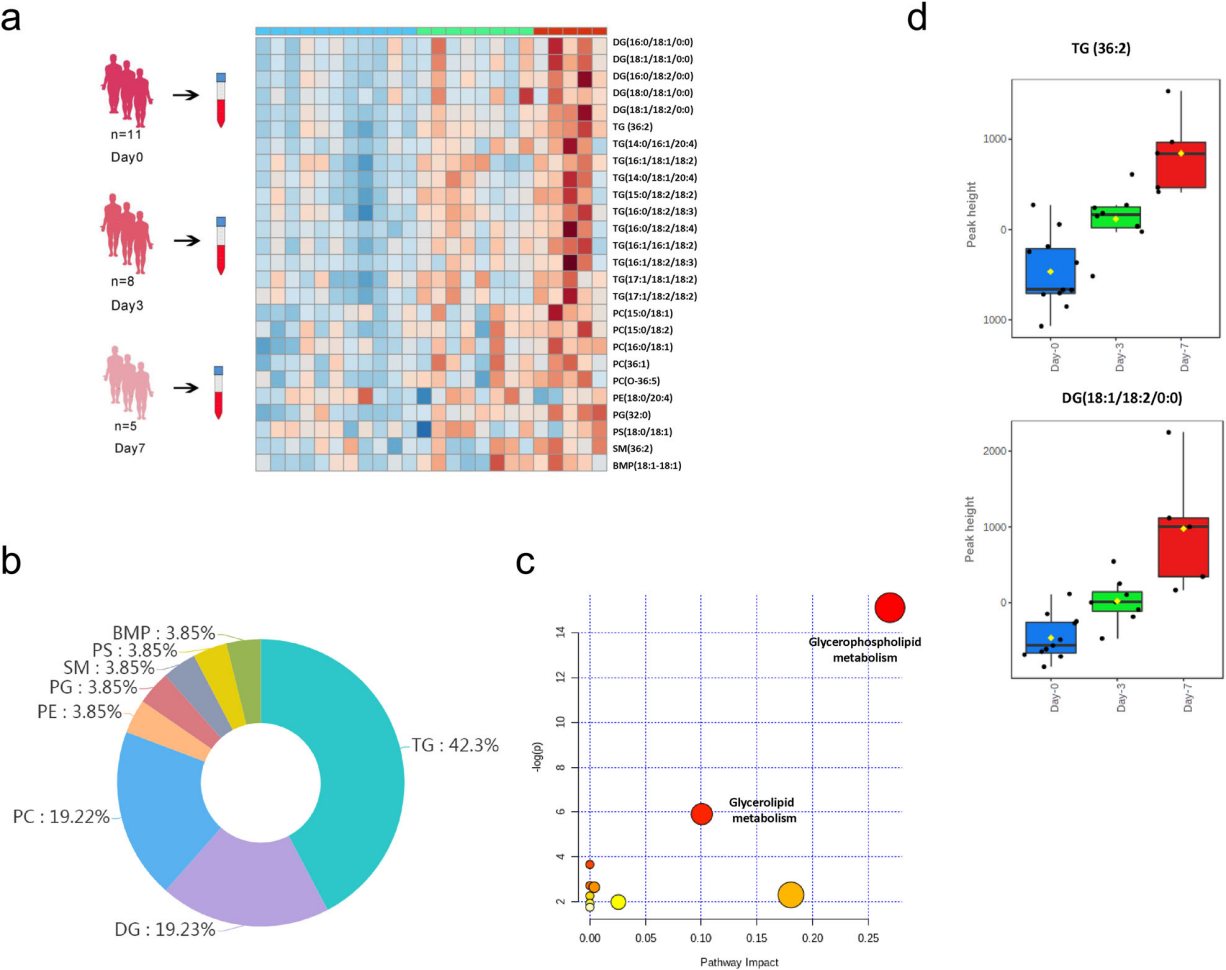
Plasma lipidome of COVID-19 patients. **a** Lipidome of COVID-19 patients’ plasma samples over the disease course. All blood samples were collected before the patients’ discharge from the hospital. Day 0 means the first day of patient hospitalization and blood taking. The hierarchical clustering analysis was based on the identified lipid metabolites with significant changes in quantity, comparing with Day 0. Each rectangle represents a lipid colored by its normalized intensity scale from blue (decreased level) to red (increased level). **b** Pie chart showing the relative ratio of eight lipid classes that are significantly perturbed. These lipids belong to BMP bismono-acylglycerophosphate. DG diacylglycerol, PC glycerophosphocholines, PE glycerophosphoethanolamines, PG glycerophosphoglycerols, PS glycerophosphoserines, SM sphingomyelin, TG triacylglycerol. **c** Overview of pathway analysis based on the identified lipids. The *y*-axis, “-log(p)”, indicates the log_10_ transformed *p*-value after enrichment analysis; the *x*-axis, “Pathway Impact”, represents the value calculated from the pathway topology analysis. **d** Boxplots illustrate the top two dominant perturbed lipid class representatives in the time-course study, DG (18:1/18:2/0:0) (*P* = 0.0097, day 0 vs day 7) and TG (36:2, day 0 vs day 7) (*P* = 0.0084). *y*-Axis represents the peak height of selected lipids based on the LC–MS data.

To depict the lipidomic landscape after SARS-CoV-2 infection over time, we monitored the lipidome of a clinical cohort with serially collected plasma specimens at days 0, 3, and 7 after hospitalization from 11 RT-qPCR-confirmed COVID-19 patients (Supplementary Table S1). All patients had mild-to-moderate symptomatic disease. A list of 350 and 172 known lipid features in the positive mode and negative mode were identified, respectively. The levels of 26 of these lipid features were significantly different between the days 0 and 7 specimens (Fig. 1a and Supplementary Table S2). Plasma lipid alterations were largely associated with increases in TG (42.31%), diacylglycerol (DG, 19.23%), and glycerophosphocholine (PC, 19.23%) (Fig. 1b), with glycerophospholipid metabolism being the most enriched pathway (Fig. 1c). Of note, the top two perturbed lipid classes, i.e., DG (18:1/18:2/0:0) and TG (36:2) were elevated during the time-course (Fig. 1d). The result indicated that the serum TG and/or DG levels increased as the disease progressed.

**Fig. 2:**
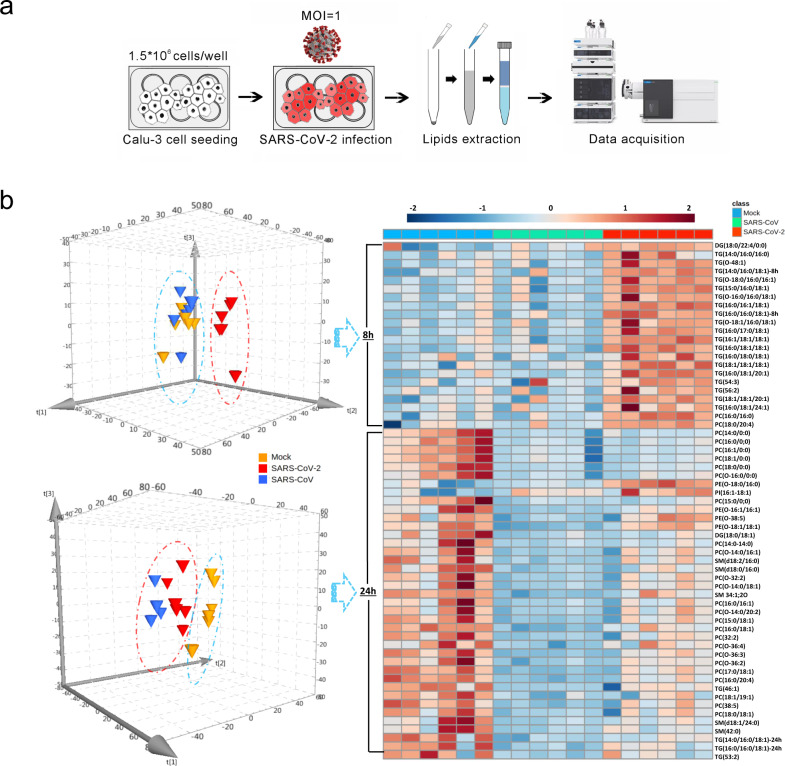
Comparative lipidome between SARS-CoV- and SARS-CoV-2- infections. **a** Schematic flow chart showing the comparative analysis between SARS-CoV- and SARS-CoV-2- infections. Human lung Calu-3 cells were infected (1 MOI) by SARS-CoV or SARS-CoV-2, followed by lipid extraction at 8 and 24 hpi, respectively. **b** Hierarchical clustering analysis was generated based on all significantly increased/decreased lipids comparing either SARS-CoV- or SARS-CoV-2- infection with that of mock infection, respectively. The 3D principal component analysis (PCA) score plots showing the distribution pattern of the detectable lipid profile, which differentiate SARS-CoV-2-infection (red) from mock- nfection (yellow) and SARS-CoV-infection (blue) groups. The triangles represent the distribution of an individual sample dot within each group (*n* = 6).

Production of pro-inflammatory cytokines such as tumor necrosis factor (TNF) and IL-6 are associated with COVID-19 severity^9^, whereas TNF is known to increase serum TG level^10^. From this perspective, the disruption of lipidomic homeostasis, as detected in patient plasma, is caused by virus-induced inflammatory stimuli. From another perspective, type II alveolar epithelial cells, the major portal of SARS-CoV-2 infection, is known to synthesize surfactant phospholipids and are essential for modulating lung function^11^. To further understand the physiological relevance of SARS-CoV-2 as a respiratory pathogen, we performed comparative lipidome profiling of human lung Calu-3 cells upon virus infection. The high multiplicity of infection (MOI) by either SARS-CoV-2 or SARS-CoV was performed, followed by lipid analysis at 8-h post-infection (hpi) and 24 hpi, respectively (Fig. 2a). In line with the observation in patient plasma, dominant upregulation of TGs was identified at 8-h post-SARS-CoV-2 infection, whereas SARS-CoV did not exhibit significant pattern change as compared to mock-infected cells (Fig. 2b and Supplementary Table S3). At 24 hpi, the lipidome profile of SARS-CoV-infected Calu-3 cells became more similar to that of SARS-CoV-2-infected than mock-infected Calu-3 cells (Fig. 2b). These results suggest that SARS-CoV-2 consistently triggers TG production in vitro and in vivo, whose synthetic pathway might be critical to fuel virus replication. Further investigation is warranted to understand the earlier lipidomic reshaping in SARS-CoV-2 than SARS-CoV infection.

### DGAT and ADRP are potential host targets for anti-SARS-CoV-2 therapy

**Fig. 3:**
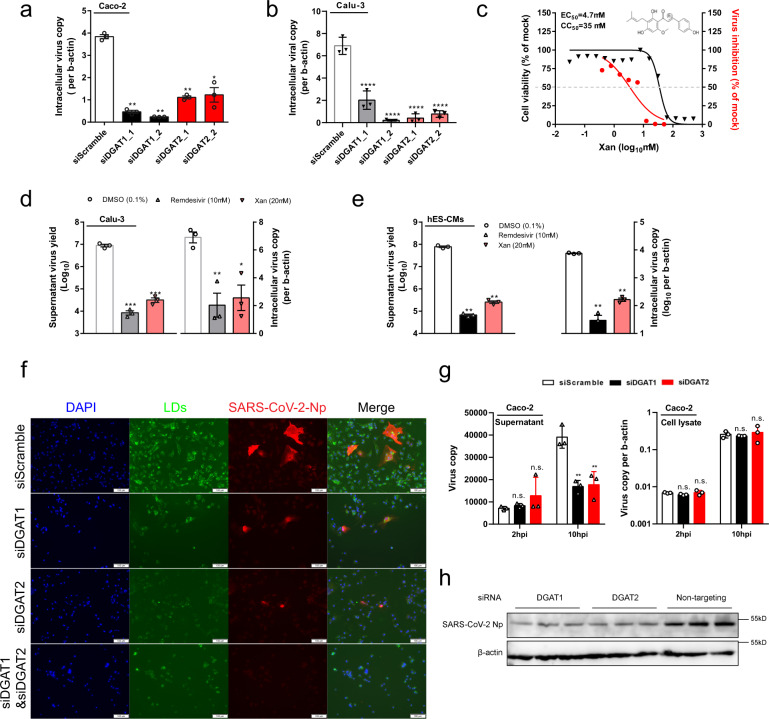
DGATs are potential therapeutic targets for COVID-19. **a**, **b** *DGAT* genes are required for SARS-CoV-2 replication. siRNA knockdown of either DGAT1 (two distinct siRNA 1_1 and 1_2) or DGAT2 (siRNA 2_1 and 2_2) were performed on human colorectal Caco-2 (**a**) or lung Calu-3 cells (**b**) before virus infection for 48 h (0.1 MOI). Viral yields in the cell lysate were determined by RT-qPCR and normalized with human β-actin. One-way ANOVA was used for comparison with the scramble siRNA pre-treated group. **c** Dose–response analysis of the compound Xanthohumol is shown, depicting both antiviral activity (red) and cytotoxicity (black). The gray dash line indicates 50% of the mock-treated control with EC_50_, CC_50_, and chemical structure displayed. **d** Xanthohumol inhibited SARS-CoV-2 replication in Calu-3 cells that were infected by 0.1 MOI SARS-CoV-2. Viral loads in the cell supernatant and cell lysate were determined at 48 hpi by RT-qPCR assays, respectively. Data represent means ± SD. One-way ANOVA was used for comparison with the DMSO control group. **e** Xanthohumol inhibited SARS-CoV-2 replication in human embryonic stem cells-derived cardiomyocytes (hES-CMs) that were infected by 0.1 MOI SARS-CoV-2. Viral loads in the cell supernatant and cell lysate were determined at 24 hpi by RT-qPCR assays, respectively. Data represent means ± SD. One-way ANOVA was used for comparison with the DMSO control group. **f** siRNA-treated-Huh7 cells were infected with SARS-CoV-2 (10 MOI for 12 h) before staining with DAPI (blue), viral nucleocapsid protein (NP) (red), and BODIPY 493/503 lipid probe (green) for LD detection. Scale bar: 100 µm. **g** Knockdown of DGAT1/2 reduced viral yields in the cell culture supernatant but not cell lysate. A single-cycle SARS-CoV-2 replication assay was performed in Caco-2 cells transfected with the indicated siRNA. Viral yields in the cell lysate and supernatant were determined by RT-qPCR. Data collected at 2 hpi were taken as a baseline and at 10 hpi taken as the completion time for one virus life cycle^2^. One-way ANOVA was used for comparison with the scrambled siRNA pre-treated group. For all statistical analyses, ^*^P < 0.05, ^**^P < 0.01, ^***^P < 0.001, ^****^P < 0.0001, n.s. indicates *P* > 0.05. **h** Cell lysate at 10 hpi was also utilized for western blotting detecting SARS-CoV-2 NP and host β-actin. Shown are triplicates (i.e., three different siRNAs) of each group.

LDs are the storage organelles accumulating TGs and cholesterol esters, which are critically involved in a wide range of virion production as the source of metabolic energy and membrane formation in flaviviruses^12^. Indeed, a considerable increase of LD accumulation was visualized in human hepatic Huh7 cells after MERS-CoV infection^6^ and human monocytes after SARS-CoV-2 infection^8^, indicating broad relevance between LDs and different viruses’ life cycles. The last step in TG synthesis is catalyzed by DGAT which esterifies the DG with a fatty acid. The two isoform DGAT1 and DGAT2 enzymes are endoplasmic reticulum-resident and have similar activities in vitro, while only DGAT2 is essential in vivo^13^. To determine if DGAT enzymes influence SARS-CoV-2 replication, siRNAs directed against DGAT1 or DGAT2 were introduced into virus-permissive human colonic Caco-2 cells with favorable knockdown efficiency. Utilizing a multi-cycle virus growth experiment, we found a significant reduction of viral yields in the lysates of siDGAT1- or siDGAT2-treated cells, with SARS-CoV-2 exhibiting generally higher dependence on DGAT1 than DGAT2 (Fig. 3a). Suppression of SARS-CoV-2 was also observed in DGAT1/2 knockdown pulmonary Calu-3 cells (Fig. 3b). The results suggest that both DGAT1 and DGAT2 are important to maintain SARS-CoV-2 replication fitness.

To investigate the druggability of targeting DGAT for anti-SARS-CoV-2 therapy, we employed a DGAT1/2 small molecule inhibitor Xanthohumol for the treatment of virus-infected Caco-2 cells. Increasing concentrations of Xanthohumol caused a dose-dependent reduction of virus titers in the cell culture supernatant and at non-toxic concentrations, with a half-maximal inhibitory concentration (EC_50_) of 4.7 ± 2.4 µM and a half cytotoxicity concentration (CC_50_) of 35 ± 5 µM (Fig. 3c). Experiments with Xanthohumol were also performed in Calu-3 cells, where significant SARS-CoV-2 viral load reduction was detected in both supernatant and cell lysate after drug compound treatment (Fig. 3d). The antiviral efficacy of Xanthohumol was also evident in human stem cell-derived cardiomyocytes, which is an established susceptible primary cell model for SARS-CoV-2 infection^14^. Importantly, cardiac complications including viral myocarditis have been increasingly reported in COVID-19 patients, and it is well recognized that synthesis and turnover of cardiac TGs, controlled by DGATs, play a pivotal role in cardiac function^15^. Indeed, we demonstrated both extracellular (~3 logs) and intracellular (~2 logs) SARS-CoV-2 viral load reduction after Xanthohumol treatment, which was in similar magnitude as that of remdesivir (Fig. 3e).

To ascertain the effects of DGAT1 and/or DGAT2 deficiency on SARS-CoV-2-induced LD formation, immunofluorescence staining was employed to visualize the intensity and abundance of LDs and viral nucleocapsid protein (NP) expression. Notably, decreased LD induction and NP expression were observed after DGAT1 or DGAT2 siRNA treatment. Double knockdown of DGAT1 and DGAT2 exhibited less LD intensity and more SARS-CoV-2 suppression when compared with either DGAT1 or DGAT2 knockdown (Fig. 3f). Next, we investigated for the exact step of the SARS-CoV-2 replication cycle that DGAT1/2 contributes to. In a single infectious cycle, we found that DGAT1/2 depletion decreased the extracellular but not intracellular viral load (Fig. 3g). Intriguingly, a reduced amount of viral NP production was also observed in the cell lysate, suggesting that DGAT1/2 affects the SARS-CoV-2 replication cycle at a stage after viral genome replication/transcription, probably at the stage of viral protein translation and/or thereafter (Fig. 3h). Taken together, DGAT is a druggable target for anti-SARS-CoV-2 intervention.

**Fig. 4:**
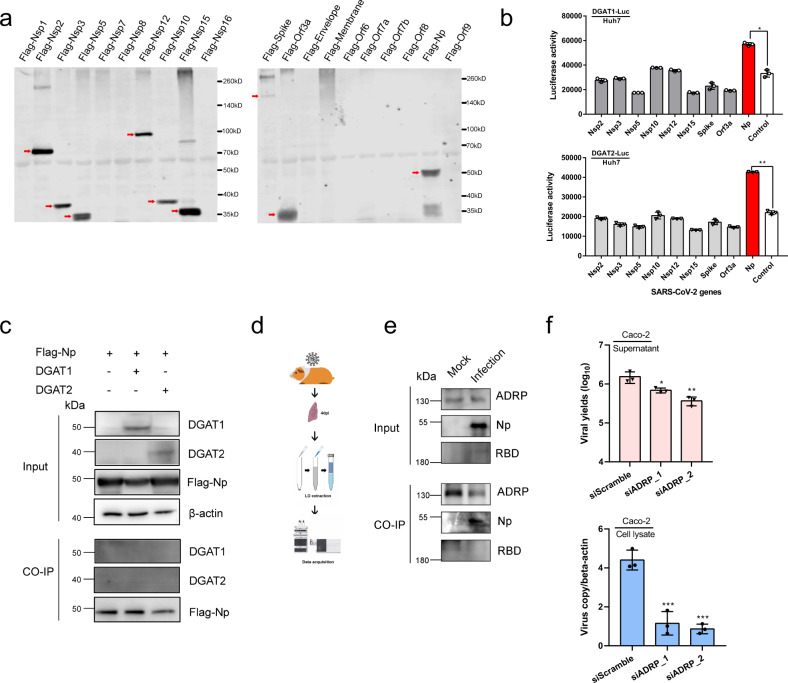
The interplay between SARS-CoV-2 and LDs-relevant host factors. **a** Expression of individual SARS-CoV-2 protein when co-transfected with the *DGAT1* or *DGAT2* promoter-reporter plasmid. Shown is the Western blot detecting the viral proteins by anti-flag antibody. **b** SARS-CoV-2-NP trans-activates *DGAT1*/*2* genes. Huh7 cells were transfected with the indicated reporter-gene plasmid and viral ORF clones, individually. The luciferase activity reflecting *DGAT1* (upper panel) or *DGAT2* (lower panel) gene expression was determined at 48 hpi. One-way AVONA was used for comparison with the control group. **c** SARS-CoV-2-NP does not interact with DGAT1 or DGAT2. A co-immunoprecipitation assay was conducted in 293 T cells transfected with NP and either DGAT1/2 plasmids. After pull-down, NP was detected by anti-flag while DGAT1/2 was detected by specific antibodies. **d** Flow chart showing the preparation of hamster lung extracts for identification of SARS-CoV-2 proteins associated with LDs. **e** ADRP interacts with viral NP but not spike. A co-immunoprecipitation assay was conducted in infected or non-infected hamster lungs. After pull-down using an anti-ADRP antibody, viral NP or spike (RBD) was detected by specific antibodies. **f** Knockdown of *ADRP* gene expression reduced SARS-CoV-2 replication in CaCo-2 cells (0.1 MOI, 48 hpi). Viral yields in the supernatant and cell lysate were determined by RT-qPCR, respectively. One-way ANOVA was used for comparison with the scrambled siRNA pre-treated group. ^***^P < 0.001, ^**^P < 0.01, ^*^P < 0.05.

To explore the interplay between SARS-CoV-2 and DGATs, we first performed a reporter gene assay detecting the DGAT transcriptional activation. A panel of SARS-CoV-2 ORF clones was co-transfected with the reporter gene plasmid carrying *DGAT1* or *DGAT2* promoter region (Fig. 4a). Among the candidate proteins, viral NP consistently enhanced both *DGAT1* and *DGAT2* gene expression whereas the other expressible viral components did not (Fig. 4b). To determine if DGAT1 and/or DGAT2 physically interacts with viral NP, co-immunoprecipitation assays were conducted, which revealed that protein–protein interaction between SARS-CoV-2 NP and DGAT1/2 was absent (Fig. 4c). These results suggest that SARS-CoV-2 NP may transcriptionally drive DGAT upregulation to meet the heightened demand of TG and LD synthesis during its replication cycle. To understand the precise impact of LD formation to the SARS-CoV-2 life cycle, we performed protein ID of viral proteins associated with the LDs. SARS-CoV-2-infected hamster lungs were harvested at 4 dpi, followed by LD isolation before BioID (Fig. 4d). Only the viral spike and NP protein fragments were consistently identified in three different hamster lungs. To validate this finding, we performed immunofluorescence staining of the virus-infected cells for visualization. Increased LDs were observed in the perinuclear region of Huh7 cells upon SARS-CoV-2 infection (Supplementary Fig. S1a). To validate the potential co-localization between NP–LDs and spike–LDs, we utilized immuno-electron microscopy to detect the antibody-conjugated gold nanoparticles in LDs. SARS-CoV-2-infected Huh7 cells were fixed before incubating with spike- or NP-antibodies. Indeed, small dark circular particles (~10 nm) were visible in LD regions of SARS-CoV-2-infected groups, which was undetectable in those of the mock-infection group (Supplementary Fig. S1b). The result indicates that viral NP and spike are associated with cellular LDs.

LDs consist of a neutral lipid core surrounded by a phospholipid monolayer containing LD-associated protein adipose differentiation-related protein (named as ADRP or PLIN2), which is the most abundant marker protein on the LDs. Critically, ADRP and LDs provide reciprocal stabilization^16^. To further investigate the interaction between the viral NP and spike with LDs, we first verified that both NP and spike were existing in the LDs extraction of SARS-CoV-2-infected hamster lung tissues using Western blotting (upper panel, Fig. 4e). Next, immunoprecipitation assays were performed, where direct interaction between ADRP and viral NP, but not spike, was observed (lower panel, Fig. 4e). Importantly, we found that siRNA knockdown of ADRP suppressed SARS-CoV-2 replication in both cell culture supernatant and lysate, suggesting the potential of targeting ADRP-governed LDs integrity for antiviral therapy (Fig. 4f). To the best of our knowledge, a direct ADRP-binding small molecule compound is not available. Therefore, screening for other repurposable drug compounds targeting the ADRP-viral–NP interaction should be considered in future studies.

### The DGAT1/2 inhibitor Xanthohumol ameliorates SARS-CoV-2 disease in a hamster model

**Fig. 5:**
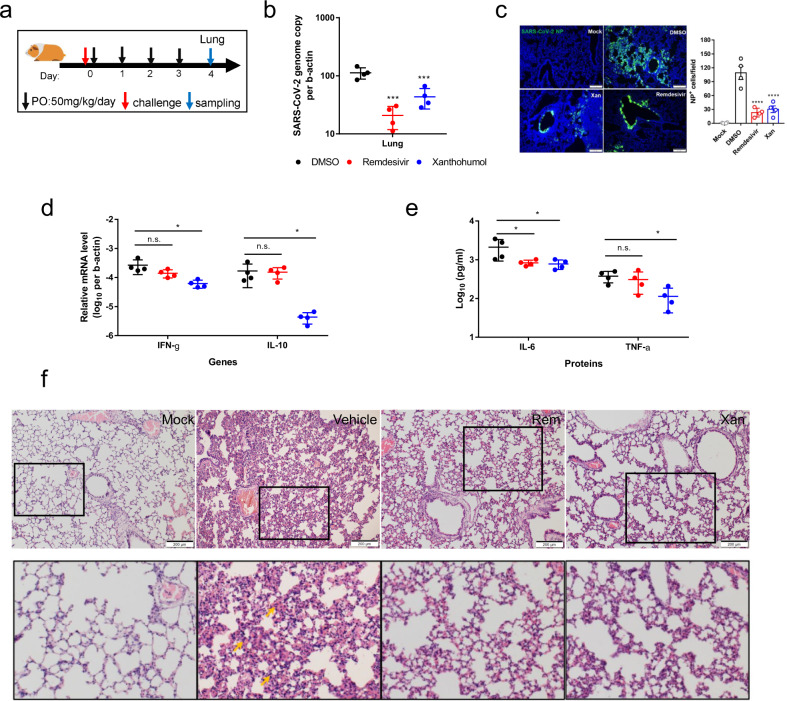
Xanthohumol inhibits SARS-CoV-2 replication in vivo. **a** Experimental design of the oral Xanthohumol regimen. **b** Viral yield in the hamster lung (*n* = 4) was harvested at 4 dpi and titrated by RT-qPCR assay. Data represent mean ± SD. One-way ANOVA was used for comparison with the DMSO control group. For all statistical analyses above, ^*^P < 0.05, ^**^P < 0.01, and ^***^P < 0.001. **c** Representative images of viral NP distribution in lung tissues section from infected hamsters treated with DMSO or Xanthohumol or Remdesivir, at 4 dpi. SARS-CoV-2 NP (green) and cell nuclei (blue) were stained. Scale bar, 200 µm. These representative images were selected from a pool of 10 images captured in 4 hamsters per group. NP-positive cells per 50× field per the lung section of a hamster were quantified. One-way ANOVA followed by Dunnett’s post test and compared with DMSO control. ^****^P < 0.0001. **d** The mRNA expression of IFN-γ and IL-10 in hamster lung was quantified at 4 dpi using qRT-PCR assays. One-way ANOVA was used for comparison with the DMSO group. **e** The cytokines IL-6 and TNF-α levels in hamster serum samples were quantified at 4 dpi using ELISA. One-way ANOVA was used for comparison with the DMSO group. **f** Representative images of H&E-stained lung tissue section from the hamsters treated with different groups indicated, illustrating the severity of alveolar infiltration (gold arrows). The lower panel are enlarged images corresponding to each black box of the upper panel. These representative images were selected from a pool of 10 images captured in 4 hamsters per group. Scale bar, 200 μm.

Next, we employed an established golden Syrian hamster model for COVID-19 to evaluate the in vivo effects of DGAT inhibition on SARS-CoV-2 infection^17^. Dorn et al.^18^ have previously reported that a high oral dose of Xanthohumol (1000 mg/kg/day for 3 weeks) causes negligible signs of toxicity in BALB/c mice, indicating a favorable drug safety profile of this natural compound found in hops (flowers of *Humulus lupulus*). A single-dose pharmacokinetic (PK) study of Xanthohumol in humans showed a half-life of 18 h^19^. In our hamster experiment, the first dose of 50 mg/kg of Xanthohumol was given via intragastric gavage at 6 hpi, followed by another 6 doses delivered from 1 to 3 dpi (twice daily with 12 h interval) (Fig. 5a). At 4 dpi when the viral loads are high and the histopathological changes are prominent in the hamsters, the animal lungs were harvested for evaluation of virological and histopathological analyses. Xanthohumol significantly reduced the SARS-CoV-2 viral load in lung tissues (*P* < 0.001, Fig. 5b). The therapeutic impact of Xanthohumol was also evidenced by the immunofluorescence staining showing markedly reduced viral NP expression in the bronchiolar and alveolar epithelia, indicating restricted virus spread (*P* < 0.0001, Fig. 5c).

Increased secretion of pro-inflammatory cytokines is associated with severe COVID-19 in human^20^. To ascertain if the therapeutic effect of Xanthohumol relieved virus-induced cytokine dysregulation, we determined the expression levels of IL-6, IL-10, and TNF-α, which are prognostic markers for severe COVID-19, as well as other major pro-inflammatory cytokines including IFN-γ. As shown in Fig. 5d, mRNA expression of IFN-γ (*P* < 0.05) and IL-10 (*P* < 0.05) were remarkably diminished in the hamsters treated with Xanthohumol, whereas those treated with remdesivir generally had lower but statistically non-significant changes compared with DMSO-treated animals. We also detected substantially decreased levels of serum IL-6 (*P* < 0.05) and TNF-α (*P* < 0.05) in Xanthohumol-treated groups (Fig. 5e). Importantly, Xanthohumol treatment also mitigated pulmonary inflammation with fewer areas of consolidation and alveolar space infiltration when compared with the DMSO control (Fig. 5f). Taken together, our data demonstrated the in vitro and in vivo effects of Xanthohumol to ameliorate SARS-CoV-2 infection and the associated inflammation.

## Discussion

**Fig. 6:**
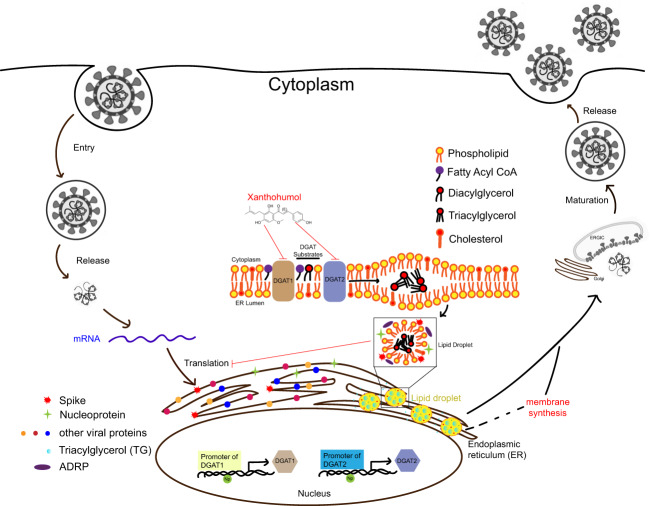
Proposed model for host DGAT and ADRP in SARS-CoV-2 life cycle. Virus manipulates host glycerophospholipid metabolism through upregulation of TG synthesis and LD formation. Specifically, viral NP trans-activates both *DGAT1* and *DGAT2* mRNA expression binding to their promoter regions, resulting in LD accumulation. Both DGATs and the other LD surface protein ADRP are required for SARS-CoV-2 replication. The identified compound Xanthohumol is a pan-DGAT1/2 inhibitor that exhibits anti-SARS-CoV-2 activity both in vitro and in vivo.

Collectively, our findings confirm the massive engagement of host LDs-networking upon SARS-CoV-2 infection and identified hitherto unrecognized roles of DGAT and ADRP during SARS-CoV-2 infection (Fig. 6). Transcriptionally, SARS-CoV-2 NP activates DGAT1/2 expression that facilitates LD formation. LDs serve as anchors for NP and spike localization, whereas the functional significance of such trafficking as well as the direct NP–ADRP interaction remains unknown at the present stage. One of the possibilities is like the case of hepatitis C virus (HCV), whose core protein has been shown to displace ADRP from the LD surface to enable efficient virus assembly^21^. In SARS-CoV-2, we found that NP upregulates DGAT1/2 expression and thus facilitates virus production; and both DGAT and ADRP determine virus fitness. Centralized via LDs, future studies are desired to elucidate the interplay among the LD-residential viral NP, the LD synthetase DGAT, and the LD stabilizer ADRP. Intriguingly, distinct lipidomic patterns were observed in cells infected with SARS-CoV-2 or SARS-CoV at 8 hpi, when SARS-CoV-2 infection markedly perturbs TG metabolism (Fig. 2). TGs serve as an important cellular energy source and are lipolytically broken down into FAs, which are then imported into mitochondria and consumed by β-oxidation to produce ATP^22^. The result indicates that SARS-CoV-2 may require higher demand of energy supply to maximally fuel its genome and protein production during the early stage of the replication cycle. Nevertheless, we cannot completely exclude the possibility that other CoVs may also perturb the host lipidome at different time points as the modulation of host lipid homeostasis is generally dynamic and the viral replication kinetics of these CoVs differ^23,24^. A number of factors, such as viral genome variance between SARS-CoV-2 and SARS-CoV, cell models used for comparison, and time-points of lipid profiling, may contribute to the apparent differences observed in our findings. Further investigations to characterize the lipidomics perturbations induced by different CoVs should be conducted.

Genetic deletion of DGAT1 in mice revealed that this enzyme is not essential and caused TG decrease in many tissues when the mice were fed with a high-fat diet^25^. Mice lacking DGAT2, however, have severe reductions in TG levels and die shortly after birth^26^. In this regard, further investigations on whether or not DGAT1^−/−^ mice are resistant to SARS-CoV-2 infection should be considered. In addition, the potential role of DGAT1 inhibition as a pan-coronavirus agent should be further explored. Increased cardiac TG content is a marker of lipotoxic cardiomyopathy, while DGAT1^−/−^ mice exhibit a number of beneficial metabolic effects^27^. On the other hand, ischemic and inflammatory responses caused by SARS-CoV-2 infection can adversely affect cardiac function. In such circumstances, Xanthohumol may potentially be effective in directly reducing SARS-CoV-2 load and virus-induced cardiac complications.

Compared with A922500, a DGAT1-specific inhibitor with documented anti-SARS-CoV-2 activity^28^, Xanthohumol inhibits both DGAT1 and DGAT2 with similar potency^29^. Since compensatory mechanisms between DGAT1 and DGAT2 exist^30^, and simultaneous suppression of DGAT1/2 activity exerts synergy when compared with that of mono-inhibition (Fig. 3f), Xanthohumol likely has a higher potential for clinical use against COVID-19 than A922500 as DGAT2 are abundantly expressed in blood, liver, and adipose tissues. In addition, Xanthohumol shows anti-inflammatory effects as evidenced by its dose-dependent inhibition of lung inflammatory infiltrate in porcine reproductive and respiratory syndrome virus infection in pigs^31^. Xanthohumol inhibits NF-κB-dependent proinflammatory gene expressions including *IL-6*^32^. Appropriate and timely inhibition of IL-6 may be beneficial in patients with severe COVID-19^33^ (Fig. 5e). Moreover, Xanthohumol can be easily extracted from hops and should therefore be more affordable than expensive antiviral drugs such as remdesivir in developing countries^19^. Importantly, in contrast to the orally available Xanthohumol, remdesivir requires intravenous administration, which makes outpatient or prophylactic use of the drug difficult. Taken together, our study identified an affordable treatment option for COVID-19. Further investigations to assess the effect of Xanthohumol in additional animal models and clinical trials should be considered.

In summary, our findings uncover that the TGs hyperproduction may partake in the metabolic dysregulation underlying COVID-19 pathogenesis and provides the basis for further development of DGAT-targeting and ADRP-targeting interventions for COVID-19 therapeutics.

## Materials and methods

### Ethics statement

All experiments involving human subjects were conducted in accordance with the Declaration of Helsinki and the International Ethical Guidelines for Biomedical Research. All donors gave written consent as approved by the Institutional Review Board of the University of Hong Kong/Hospital Authority Hong Kong West Cluster (Reference code: UW13-364). All animal experimental protocols were approved by the Animal Ethics Committee in the University of Hong Kong (CULATR) and were performed according to the standard operating procedures of the biosafety level 3 animal facilities (Reference code: CULATR 5370-20). All experiments involving live SARS-CoV-2 and SARS-CoV followed the approved standard operating procedures of the Biosafety Level 3 facility at the Department of Microbiology, The University of Hong Kong^34,35^.

### Cells and viruses

Human cardiomyocytes were differentiated from the human embryonic stem cell HES2 (ESI) maintained in mTeSR1 medium (STEMCELL Technologies), as previously described^36^. Briefly, HES2 cells were dissociated with Accutase (Invitrogen) into single cells suspension on day 0. Cells were seeded on low-attachment culture vessels (Corning) and cultured in mTeSR1 medium supplemented with 40 µg/mL Matrigel, 1 ng/mL BMP4 (Invitrogen), and 10 µM Rho kinase inhibitor (ROCK) (R&D) under the hypoxic environment with 5% O_2_. From day 1 to 3, cells were cultured in StemPro34 SFM (Invitrogen) with 50 µg/mL ascorbic acid (AA) (Sigma), 2 mM Gluta-MAX (Invitrogen), 10 ng/mL BMP4, and 10 ng/mL human recombinant activin-A (Invitrogen). From day 4 to day 7, 5 µM Wnt inhibitor IWR-1, a Wnt inhibitor (Tocris) was added. From day 8 to day 14, cells were cultured under normoxia in RPMI 1640 medium (Invitrogen) supplemented with 2 mM Gluta-MAX, 1× B-27 supplement (Invitrogen), and 50 µg/mL AA. The cells were then dissociated with Accutase and seeded as monolayers in desired culture vessels for 3 days before infections. Vero-E6 (ATCC^®^ CRL-1586^™^), Calu-3(ATCC^®^ HTB-55^™^), and human hepatoma (Huh7, JCRB^®^ 0403^™^) cells were maintained in Dulbecco’s modified eagle medium (DMEM, Gibco, CA, USA) culture medium supplemented with 10% heat-inactivated fetal bovine serum (FBS, Gibco), 50 U/mL penicillin, and 50 μg/mL streptomycin. All cell lines were confirmed to be free of mycoplasma contamination by Plasmo Test (InvivoGen). SARS-CoV-2 HKU-001a (GenBank accession number: MT230904) was isolated from the nasopharyngeal aspirate specimen of a laboratory-confirmed COVID-19 patient in Hong Kong; SARS-CoV (GZ50 strain) was kept in our lab previously^37^. The viruses were propagated in Vero-E6 cells and kept at −80 °C in aliquots until use. Plaque forming units (PFU) were performed to titrate the cultured SARS-CoV-2 and SARS-CoV^38^.

### Plasma collection and lipid extraction

Patient blood was collected by regular phlebotomy directly into BD Vacutainer^®^ CPT^™^ tubes (heparin-Ficoll tubes). Plasma was separated by centrifugation at 3000 rpm for 20 min. Lipid extraction was performed for liquid chromatography–mass spectrometry (LC–MS) analysis as we previously described with slight modifications^39^. Briefly, 20 µL ice-cold methanol contained internal standards and butylated hydroxytoluene (BHT) was added into 50 µL of each plasma sample. After vortex for 5 s, sample mixtures were placed on ice for 5 min before vortex again for another 30 s. After that, samples were incubated for 5 min at 1500 rpm at 4 °C in an orbital mixer before centrifugation at 4500 rpm for 10 min at 4 °C. The lipid-containing bottom phase was transferred to glass vials and lyophilized using Centrivap cold trap (Labconco) and stored at −80 °C before analysis.

### Cell culture-based lipidomic sample preparation

Calu-3 cells in a 6-well plate were mock-infected or infected with SARS-CoV-2 or SARS-CoV at 1 MOI. At 8 hpi, cells were collected for cellular lipid extraction using the protocol as we previously described^37^. Briefly, 550 µL of ice-cold 150 mM ammonium bicarbonate solution was added to dissociate cells and 50 µL of cell suspension was used for DNA extraction for normalization. A total of 250 µL of methanol containing internal standards and BHT was added to the wells. Then, 2 mL of chloroform/methanol (v/v 2:1) was added, followed by vortexing and centrifugation at 4500 rpm for 10 min at 4 °C. The bottom phase was transferred to glass vials and freeze-dried before storing at −80 °C.

### LC–MS based untargeted lipidomics

Untargeted lipidomic analysis was performed. The lipid samples were analyzed using an Acquity UPLC system coupled to a Synapt G2-Si high definition mass spectrometry system (Waters Corp., Milford, MA, USA). The chromatography was performed on a Waters ACQUITY CSH C18 column (100 × 2.1 mm; 1.7 µm) coupled to an Acquity CSH C18 VanGuard pre-column (5 × 2.1 mm; 1.7 µm) (Waters; Milford, MA, USA). The column and autosampler temperature were maintained at 55 and 4 °C, respectively. The injection volume was 7 µL for negative and 5 µL for positive. The mass spectrometer was operated in MS^E^ mode and the data were acquired in both positive and negative modes. The capillary voltage was maintained at 2.5 kV (positive mode) and 2.0 kV (negative mode). Mass spectral data were acquired over the m/z range of 100–1500. MS/MS acquisition was operated in the same parameters as in MS acquisition. The collision energy was applied at the range from 30 to 45 eV for fragmentation to allow putative identification and structural elucidation of the significant lipids.

A total of 15 lipid internal standards were applied for sample preparation and LC–MS analysis for monitoring the lipids coverage and extraction efficiency including of Arachidonic acid-d8, Platelet-activating factor C-16-d4 (PAF C-16-d4), 22:1 Cholesterol Ester, PE (17:0/17:0), PG (17:0/17:0), PC (17:0/0:0), C17 Sphingosine, C17 Ceramide, SM (d18:1/17:0), PC (12:0/13:0), Cholesterol (d7), TG (17:0/17:1/17:0) d5, DG (12:0/12:0/0:0), DG (18:1/2:0/0:0), PE (17:1/0:0) and commercial standards used for lipids identification. They all were purchased from Cayman Chemical (Ann Arbor, MI, USA) and Avanti Polar Lipids, Inc (Alabaster, AL, USA). Quality control (QC) samples were pooled and prepared by mixing equal aliquots of all of the biological samples for evaluating data acquisition.

### Data processing, statistical analysis, and lipids identification

The lipidomic data were processed to the data matrix by the MS-DIAL software before further statistical analysis. MetaboAnalyst 4.0 (http://www.metaboanalyst.ca) and SIMCA-P V12.0 (Umetrics, Umeå, Sweden) were used for univariate and multivariate analysis, respectively. QC and DNA-based normalization methods were applied during data preprocessing. For human subject samples, the FDR- adjust *P*-value < 0.05 and fold change > 1.5 or < 0.67 were set as a criterion for selecting significant features. For Calu-3 cell-based samples, FDR adjust *P*-value < 0.05 and fold change > 1.25 or < 0.8 used as a cut-off. In multivariate analysis, the partial least squares discriminant analysis was applied to find important variables with discriminative power.

The significant lipid features were identified by searching accurate MS and MS/MS fragmentation pattern data in the MS-DIAL internal lipid database, MassBank of North America (MoNA, http://mona.fiehnlab.ucdavis.edu/), METLIN database (http://metlin.scripps.edu/), and LIPID MAPS (http://www.lipidmaps.org/). For confirmation of lipid identity using authentic chemical standards, the MS/MS fragmentation patterns of the chemical standards were compared with those of the candidate lipids measured under the same LC–MS condition. Pathway analysis was performed by Metaboanalyst and KEGG mapper.

### Animal model

Male and female Syrian hamsters, aged 6–10 weeks old, were kept in biosafety level housing and given access to standard pellet feed and water ad libitum as we previously described^17^. Hamsters were randomly allocated to experimental groups (*n* = 4) for antiviral evaluation. No blinding was applied. All experimental protocols were approved by the Animal Ethics Committee in the University of Hong Kong (CULATR) and were performed according to the standard operating procedures of the biosafety level 3 animal facilities (Reference code: CULATR 5370-20)^40^. Experimentally, each hamster was intranasally inoculated with 10^4^ PFU of SARS-CoV-2 in 100 µL phosphate-buffered saline (PBS) under intraperitoneal ketamine (200 mg/kg) and xylazine (10 mg/kg) anesthesia. Oral administration of Xanthohumol was started at 6 hpi (50 mg/kg/day) and then continued for 6 additional doses (each dose with 12 h interval) from 1 to 3 dpi. Xanthohumol was delivered using corn oil (Sigma-Aldrich, C8267) as a vehicle. One percent DMSO in corn oil was taken as a placebo control through the oral route. Animals were sacrificed at 4 dpi for virological analyses. Lung tissue samples were collected for virological investigations. Viral yield in the tissue homogenates was detected by the qRT-PCR method. IL-6 and TNF-α expression in hamster serum was quantified by ELISA kits purchased from the AssayGenie company. Histopathological analyses and immunofluorescence staining of fixed lung tissues were performed as we previously reported^41^.

### LD extraction and protein ID

To identify the viral protein(s) associated with LDs, hamster lungs on 4 dpi were subjected to LD extraction utilizing the manufacturer’s protocol (Cell Biolabs, Cat# MET-5011). Healthy hamster lung without infection was taken as a negative control. The LD maker ADRP was included as a positive control protein within the LD extractions. The LD extraction of each infected hamster lung was sent to the Center for PanorOmic Sciences (CPOS) of the University of Hong Kong for viral protein identification. Protein Identification is performed on an online reverse-phase nanoLC coupled to an Orbitrap Fusion Lumos mass spectrometer. The SEQUEST search engine is used to search the acquired MS/MS spectra, the resulting protein identifications are further validated using the Proteome Discoverer (PD) software.

### Cytotoxicity and antiviral measurement in vitro

The CellTiterGlo^®^ luminescent assay (Promega Corporation, Madison, WI, USA) was performed to detect the cytotoxicity of the selected drug compounds as previously described^42^. The half cytotoxicity concentration (CC_50_) of the drug was calculated by Prism7 (GraphPad). A viral load reduction assay was performed on the indicated cells. Supernatant samples from the infected cells were harvested for qRT-PCR analysis of virus replication^2^. To plot the half effective concentration (EC_50_) of Xanthohumol, the collected supernatant was subject to standard plaque assay on Vero-E6 cells and calculated by Prism7 (GraphPad).

### Immunofluorescence staining

Huh7 cells in an 8-well chamber slide (LAB-TEK) were infected with SARS-CoV-2 (10 MOI) for 12 h before fixation with 4% Paraformaldehyde. After blocking, SARS-CoV-2 NP was detected using the in-house rabbit anti-SARS-CoV-2 NP serum (in house, 1:500) and Alexa Fluor 594 goat anti-rabbit IgG (H + L) antibody (Abcam, ab150080, 1:500). Cells were stained with DAPI and BODIPY 493/503 lipid probe (Invitrogen) for visualization of nucleus and LDs, respectively. The confocal imaging was taken by Carl Zeiss LSM 800 microscope.

### Immunoelectron microscopy

Huh7 cells were grown in six-well plates. The cell culture medium was removed after infection with SARS-CoV-2 at 10 MOI for 24 h. The cells were washed with PBS, trypsinized, fixed with 4% PFA before further processing. Ultra-thin sections (10 nm) were cut and mounted on nickel grids. Grids were incubated in a blocking solution containing 1% bovine serum albumin and 0.1% PBS for 1 h and subsequently cross-reacted with rabbit anti-NP serum (in house, 1:50) or rabbit-anti-RBD serum (in house, 1:50) at 4 °C overnight. After intensive washing, grids were incubated in 1:100 dilution of anti-rabbit antibodies conjugated to 10 nm gold (Sigma) at room temperature for 1 h. Finally, the grids were stained with 2% uranyl acetate for 2 min before visualization. The images were acquired in a Philips CM100 Transmission Electron Microscope located in the Electron Microscope Unit of the University of Hong Kong^37^.

### Reporter gene assay

*DGAT1* and *DGAT2* promoter reporter clones were purchased from GeneCopoeia (Cat# HPRM47226-PG02 and HPRM39535-PG02, respectively). SARS-CoV-2 ORF clones were constructed as we previously described^43^. All gene fragments were cloned into pCAGEN expression vector with C-terminal FLAG-tag and confirmed by Sanger sequencing. Huh7 cells were transfected with the indicated reporter plasmid and each SARS-CoV-2 ORF clone, individually. Gaussia luciferase activity was determined at 48 h post-transfection using the Gaussia luciferase flash assay kits (Pierce). Nano-Luc was included as an internal control for normalization of cell viability and transfection efficiency.

## Supplementary information

Supplementary materials

Chemical structure of Xanthohumol

## Data Availability

All data have been presented in the main text and supplementary information of this paper.
