# Supplementary material for: SARS-CoV-2 exploits host DGAT and ADRP for efficient replication
Source: Cell Discov. 2021 Oct 26;7:100. doi: 10.1038/s41421-021-00338-2 (PMC8548329; doi:10.1038/s41421-021-00338-2)
Supplement: Supplementary file 1 — Supplementary materials [file 41421_2021_338_MOESM1_ESM.pdf]

Supplementary information

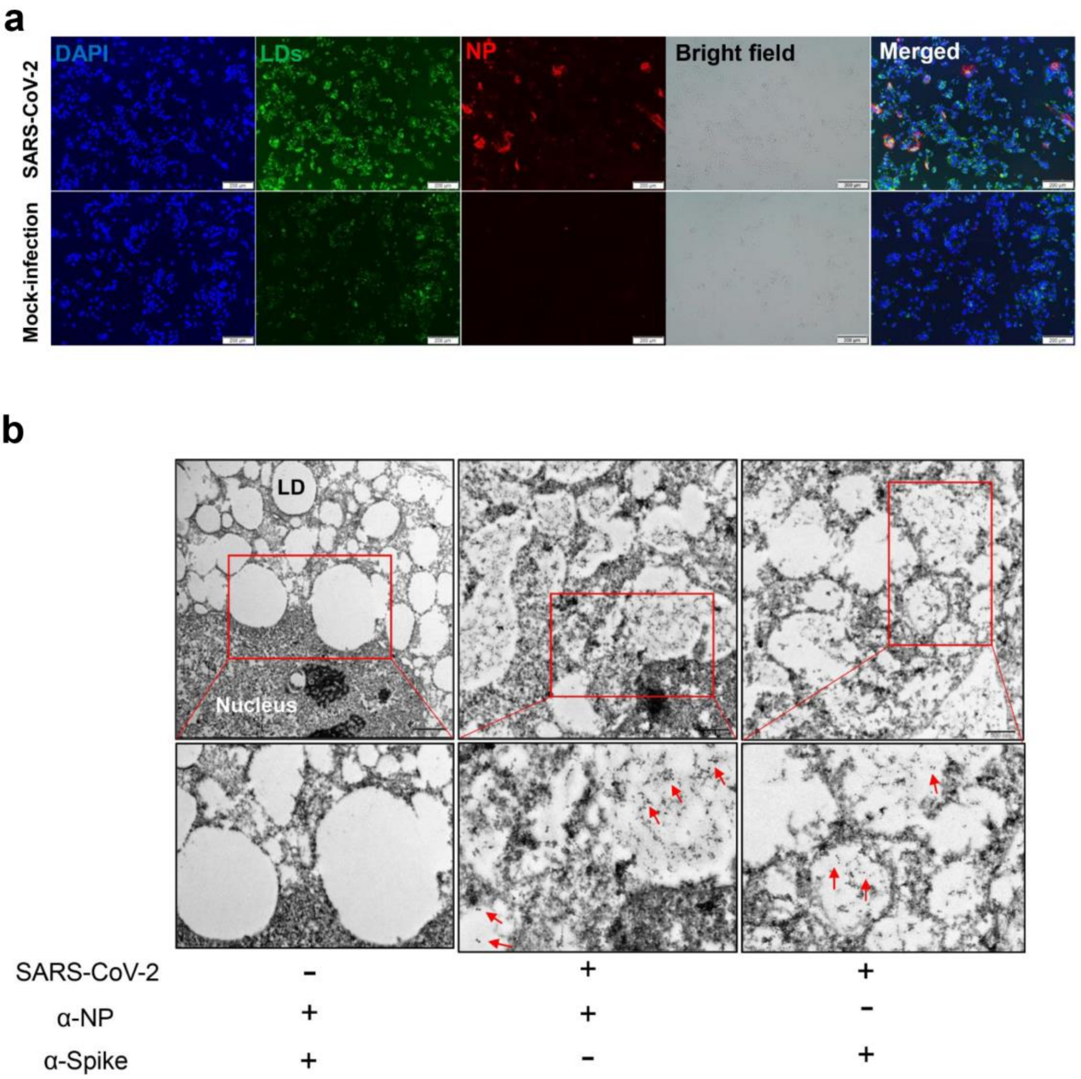

Supplementary Fig. S1. LDs increases upon SARS-CoV-2 infection. (a) SARS-CoV-2-infected Huh7 cells (10 MOI for 12h) were stained with DAPI (blue), viral antigen NP and BODIPY 493/503 lipid probe (green) for LDs detection. Scale bar: 200 µm. (b) Immunoelectron microscopy images showing of SARS-CoV-2 NP and spike localization in cellular LDs. Red arrows indicate sites of gold particles that reacted with anti-NP or anti-spike antibodies. Scale bar: 500 nm.

Supplementary Table S1: Demographic and clinical information of the COVID-19 patients in this study.

Patient	Sex	Age (years)	Obese	Comorbidities	Hospital stay (days)
**1**	Male	84	No	None	10
**2**	Female	57	No	None	15
**3**	Female	52	No	None	13
**4**	Female	48	No	None	23
**5**	Male	69	No	Hypertension, diabetes mellitus, hyperlipidemia	32
**6**	Female	30	No	History of thyroiditis	16
**7**	Female	30	No	None	12
**8**	Male	59	No	None	14
**9**	Male	32	No	None	33
**10**	Male	39	No	None	14
**11**	Male	29	No	None	14

Supplementary Table S2 | The identified lipids that were statistically different between Days 0 and 7 of COVID-19 patients' plasma.

Lipid name	Detection mode	Retention time	Accurate mass in detection mode	Adduct ion name	lipids species	p-Value	Fold Change (day 7 vs day 0)
DG(16:0/18:1/0:0)	positive	13.54	612.5684	[M+NH4]+	DG	0.0412	2.27
DG(18:1/18:1/0:0)	positive	13.59	638.5765	[M+NH4]+	DG	0.0264	2.66
DG(16:0/18:2/0:0)	positive	13.03	610.5460	[M+NH4]+	DG	0.0248	2.76
DG(18:0/18:1/0:0)	positive	14.07	640.5920	[M+NH4]+	DG	0.0420	2.16
DG(18:1/18:2/0:0)	positive	13.11	636.5610	[M+NH4]+	DG	0.0097	3.06
TG(36:2)	positive	13.11	657.4916	[M+Na]+	TG	0.0084	2.00
TG(14:0/16:1/20:4)	positive	15.24	842.7287	[M+NH4]+	TG	0.0412	2.61
TG(16:1/18:1/18:2)	positive	15.83	872.7899	[M+NH4]+	TG	0.0394	1.61
TG(14:0/18:1/20:4)	positive	15.56	870.7596	[M+NH4]+	TG	0.0356	2.30
TG(15:0/18:2/18:2)	positive	15.67	858.7743	[M+NH4]+	TG	0.0356	2.06
TG(16:0/18:2/18:3)	positive	15.56	875.7296	[M+Na]+	TG	0.0356	1.78
TG(16:0/18:2/18:4)	positive	15.31	873.7138	[M+Na]+	TG	0.0356	3.33
TG(16:1/16:1/18:2)	positive	15.51	844.7457	[M+NH4]+	TG	0.0402	2.33
TG(16:1/18:2/18:3)	positive	15.31	868.7576	[M+NH4]+	TG	0.0402	3.63
TG(17:1/18:1/18:2)	positive	15.95	886.8062	[M+NH4]+	TG	0.0474	1.56
TG(17:1/18:2/18:2)	positive	15.68	884.7887	[M+NH4]+	TG	0.0356	1.81
PC(15:0/18:1)	positive	8.63	746.5857	[M+H]+	PC	0.0395	1.76
PC(15:0/18:2)	positive	6.88	744.5609	[M+H]+	PC	0.0394	1.68
PC(16:0/18:1)	positive	9.97	760.5902	[M+H]+	PC	0.0394	1.51
PC(36:1)	positive	12.96	810.6152	[M+Na]+	PC	0.0474	1.53
PC(O-36:5)	positive	6.91	766.5423	[M+H]+	EtherPC	0.0356	1.74
PE(18:0/20:4)	negative	11.99	766.5399	[M-H]-	PE	0.0331	1.94
PG(32:0)	positive	8.23	740.5294	[M+H]+	PG	0.0212	2.32
PS(18:0/18:1)	negative	7.46	788.5450	[M-H]-	PS	0.0331	1.66
SM(36:2)	positive	7.39	751.5723	[M+Na]+	SM	0.0460	1.62
BMP(18:1/18:1)	positive	6.17	792.5607	[M+H]+	BMP	0.0356	1.80

Supplementary Table S3 | The identified lipids in Calu-3 cells that were statistically different with or without SARS-CoV-2 infection at 8hpi.

**Lipid name**	**Detection mode**	**Retention time**	**Accurate mass in detection mode**	**Adduct ion name**	**lipids species**	**p-Value**	**Fold change (SARS-CoV-2 vs Mock-infection)**
DG(18:0/22:4/0:0)	positive	13.203	690.60486	[M+NH4]+	DG	0.0272	1.48
TG(14:0/16:0/16:0)	positive	15.635	796.73853	[M+NH4]+	TG	0.0305	1.35
TG(14:0/16:0/18:1)	positive	15.635	827.71008	[M+Na]+	TG	0.0041	1.32
TG(15:0/16:0/18:1)	positive	15.787	836.77014	[M+NH4]+	TG	0.0038	1.26
TG(16:0/16:0/18:1)	positive	15.954	850.7851	[M+NH4]+	TG	0.0032	1.30
TG(16:0/16:1/18:1)	positive	15.632	848.76953	[M+NH4]+	TG	0.0032	1.25
TG(16:0/17:0/18:1)	positive	16.086	864.81598	[M+NH4]+	TG	0.0057	1.32
TG(16:0/18:0/18:1)	positive	16.239	878.83643	[M+NH4]+	TG	0.0467	1.27
TG(16:0/18:1/18:1)	positive	15.944	876.80017	[M+NH4]+	TG	0.0022	1.30
TG(16:1/18:1/18:1)	positive	15.64	874.78479	[M+NH4]+	TG	0.0032	1.26
TG(16:0/18:1/20:1)	positive	16.222	904.84778	[M+NH4]+	TG	0.0076	1.37
TG(54:3)	positive	15.921	907.78925	[M+Na]+	TG	0.0053	1.39
TG(18:1/18:1/18:1)	positive	15.93	902.8161	[M+NH4]+	TG	0.0095	1.30
TG(56:2)	positive	16.482	932.87732	[M+NH4]+	TG	0.0381	1.33
TG(18:1/18:1/20:1)	positive	16.199	935.81873	[M+Na]+	TG	0.0048	1.39
TG(16:0/18:1/24:1)	positive	16.729	960.91064	[M+NH4]+	TG	0.0425	1.34
TG(O-48:1)	positive	16.086	808.77521	[M+NH4]+	EtherTG	0.0048	1.32
TG(O-16:0/16:0/18:1)	positive	16.373	836.80756	[M+NH4]+	EtherTG	0.0100	1.37
TG(O-18:0/16:0/16:1)	positive	16.081	834.79102	[M+NH4]+	EtherTG	0.0048	1.31
TG(O-18:1/16:0/18:1)	positive	16.353	862.82074	[M+NH4]+	EtherTG	0.0102	1.36
PC(16:0/16:0)	positive	7.077	734.56879	[M+H]+	PC	0.0048	1.27
PC(18:0/20:4)	positive	7.361	810.60889	[M+H]+	PC	0.0232	1.37
